# Supplementary material for: Interpretation of vulnerability and cumulative disadvantage among unaccompanied adolescent migrants in Greece: A qualitative study
Source: PLoS Med. 2020 Mar 27;17(3):e1003087. doi: 10.1371/journal.pmed.1003087 (PMC7100937; doi:10.1371/journal.pmed.1003087)
Supplement: S1 Text — (DOCX) [file pmed.1003087.s002.docx]

## Key Informant Interview Guide

Introduction for study participant

- Thank you for agreeing to give an interview for this study.
- First, I would to ask a few questions about your experiences working in child protection.

Questions

1. Can you tell me about your current work in child protection? *Probe for:*
   1. What their role within that organization is
   2. What the key informants’ day-to-day responsibilities with respect to child protection are
   3. What are interactions with unaccompanied minors like?
   4. What are interactions with other NGOs like?
2. Can you tell me about how you came to be involved in child protection? *Probe for:*
   1. How did the key informant get started working in child protection?
   2. What previous work experiences may have prepared the key informant for their current work?
   3. What other child protection programs did they work for in the past, if any?
   4. What other humanitarian assistance programs did they work for in the past, if any?
3. What are the typical ages and nationalities of the unaccompanied minors you work with?
4. What are some of the challenges these unaccompanied minors face while living in child protection?
5. What are some of the long-term challenges these unaccompanied minors might face in over the course of their lives?
6. Many unaccompanied minors are older teenagers who will soon turn 18. What are some challenges that these young people might face after turning 18? *Probe for:*
   1. Concerns or challenges that older unaccompanied minors have when they’re about to turn 18.
   2. Concerns or challenges that older unaccompanied minors have after they turn 18.
7. How does the organization you work for respond to the needs of unaccompanied minors as they transition into adulthood?
   1. What do staff in your organization do to help prepare asylum seekers for living independently once they are 18?
8. What kind of contact do former unaccompanied minors have with the child protection program they lived in after they turn 18?
9. What is your perspective on the role that humanitarian child protection programs can play with respect to unaccompanied minors’ transition into adulthood?
