# Supplementary material for: Interpretation of vulnerability and cumulative disadvantage among unaccompanied adolescent migrants in Greece: A qualitative study
Source: PLoS Med. 2020 Mar 27;17(3):e1003087. doi: 10.1371/journal.pmed.1003087 (PMC7100937; doi:10.1371/journal.pmed.1003087)
Supplement: S2 Text — (DOCX) [file pmed.1003087.s003.docx]

## In-depth Interview Guide

Introduction for Study Participant

- Thank you for agreeing to give an interview for this study.
- Throughout this interview, we will ask you to mark important events in your life on this line in the order that they happened. Marking down major events will help me ask questions about things that happened in your life.

Questions:

1. Can you tell me how you happened to come to Greece?
   1. What year did you leave [home country]? *[mark on life history calendar]*
   2. How old were you when you left? *[mark on life history calendar]*
2. Can you tell me about what happened along your journey?
   1. How long did you stay in [transit country/particular living circumstance]? *[mark on life history calendar]*
   2. What were your thoughts/feelings regarding [events/circumstances described]?
   3. Did anyone ever harass or harm you along the journey?
   4. Who paid for your journey?
   5. Did you have a cell phone when you traveled?
   6. Did anything happen along the journey that stands out?
   7. Then what happened? [ask as many times as needed until interviewee describes arrival in Greece]
3. When did you arrive in Greece? *[mark on life history calendar]*
   1. How old were you when you arrived in Greece? *[mark on life history calendar]*
   2. Where did you live when you arrived in Greece? *[mark on life history calendar]*
   3. Did you live with adults or only boys your age?
   4. Did anyone ever try to harass or harm you?
   5. How long did you stay in [particular living circumstance]? *[mark on life history calendar]*
   6. What were your thoughts/feelings regarding [events/circumstances described]?
   7. Then what happened? [ask as many times as needed until interviewee’s narrative reaches the present]
4. Did you receive help from any NGOs in Greece?
   1. What NGOs?
   2. What kind of help and for how long? *[mark on life history calendar]*
   3. What are your thoughts regarding this aid?
5. Do you currently receive help from NGOs?
   1. What NGOs?
   2. What kind of help and for how long? *[mark on life history calendar]*
   3. What are your thoughts regarding this aid?
6. Have you ever tried to find work?
   1. Where? When? What happened?
   2. Who connected you to this work?
   3. Had you done this kind of work before? If so, when?
   4. Did anyone ever try to harass or harm you?
7. What do you do to make sure you have enough to eat, a place to shower, and other basic needs?
   1. How did you find these resources?
   2. Is there anything else you do to get extra cash?
8. Do you talk to your family back home?
   1. How often?
   2. What do you tell them about?
9. Where do you currently live?
   1. How long have you lived there?
   2. Who lives with you?
10. Do you have and European friends?
    1. Describe who they are.
    2. How did you get to know them?
11. Do you speak English or Greek?
    1. Where and when did you learn [language]?
12. Do you have asylum/white card?
    1. What does the white card mean?
    2. When did you apply for asylum? [gauge details of where interviewee is in application process; *mark on life history calendar*]
    3. Who helped you apply for asylum?
13. What are your plans for the future?
