# Supplementary material for: Interpretation of vulnerability and cumulative disadvantage among unaccompanied adolescent migrants in Greece: A qualitative study
Source: PLoS Med. 2020 Mar 27;17(3):e1003087. doi: 10.1371/journal.pmed.1003087 (PMC7100937; doi:10.1371/journal.pmed.1003087)
Supplement: S3 Text — (DOCX) [file pmed.1003087.s004.docx]

## Life History Calendar for follow-up interview guide

| Living Arrangements | | | | Age | Needs and Motivations | | | |
| --- | --- | --- | --- | --- | --- | --- | --- | --- |
| Where did you live? | What kind of house? | Who were you living with? | Who supports you (social and economic)? |  | Important needs | Challenges/  difficulties | Major  Decisions | Goals |
|  |  |  |  | 0-11 mo. |  |  |  |  |
|  |  |  |  | 1 |  |  |  |  |
|  |  |  |  | 2 |  |  |  |  |
|  |  |  |  | 3 |  |  |  |  |
|  |  |  |  | 4 |  |  |  |  |
|  |  |  |  | 5 |  |  |  |  |
|  |  |  |  | 6 |  |  |  |  |
|  |  |  |  | 7 |  |  |  |  |
|  |  |  |  | 8 |  |  |  |  |
|  |  |  |  | 9 |  |  |  |  |
|  |  |  |  | 10 |  |  |  |  |
|  |  |  |  | 11 |  |  |  |  |
|  |  |  |  | 12 |  |  |  |  |
|  |  |  |  | 13 |  |  |  |  |
|  |  |  |  | 14 |  |  |  |  |
|  |  |  |  | 15 |  |  |  |  |
|  |  |  |  | 16 |  |  |  |  |
|  |  |  |  | 17 |  |  |  |  |
|  |  |  |  | 18 |  |  |  |  |
|  |  |  |  | 19 |  |  |  |  |
|  |  |  |  | 20 |  |  |  |  |
|  |  |  |  | 21 |  |  |  |  |

Probe for details regarding financial and educational background.
