# Supplementary material for: Interpretation of vulnerability and cumulative disadvantage among unaccompanied adolescent migrants in Greece: A qualitative study
Source: PLoS Med. 2020 Mar 27;17(3):e1003087. doi: 10.1371/journal.pmed.1003087 (PMC7100937; doi:10.1371/journal.pmed.1003087)
Supplement: Attachment — Submitted filename: PMEDICINE-D-19-03759 mishra ref3.pdf [file pmed.1003087.s005.pdf]

PLOS Medicine

"Neither minor, but not yet adults": Interpretations of vulnerability and cumulative disadvantage for unaccompanied adolescent migrants in Greece

--Manuscript Draft--

Manuscript Number:	PMEDICINE-D-19-03759
Full Title:	"Neither minor, but not yet adults": Interpretations of vulnerability and cumulative disadvantage for unaccompanied adolescent migrants in Greece
Short Title:	Vulnerability and cumulative disadvantage of unaccompanied minors in Greece
Article Type:	Research Article
Keywords:	Asylum seekers; unaccompanied minors; adolescent development; vulnerability; prolonged journeys; cumulative disadvantage; shelter; child protection; Greece
Corresponding Author:	Divya Mishra, PhD<br>Johns Hopkins University Bloomberg School of Public Health<br>Baltimore, MD UNITED STATES
Corresponding Author Secondary Information:	
Corresponding Author's Institution:	Johns Hopkins University Bloomberg School of Public Health
Corresponding Author's Secondary Institution:	
First Author:	Divya Mishra, PhD
First Author Secondary Information:	
Order of Authors:	Divya Mishra, PhD
	Paul B. Spiegel
	Vasileia Lucero Digidiki
	Peter J. Winch
Order of Authors Secondary Information:	
Abstract:	Background: In settings of mass displacement, unaccompanied minors (UAMs) are recognized as a vulnerable group and consequently prioritized by relief efforts.This study examines how the operationalization of vulnerability by the national shelter system for male UAMs in Greece shapes their trajectories into adulthood.<br>Methods and Findings: In 2018-2019, key informant interviews were carried out in organizations that refer UAMs to specialized shelters in Athens to understand how child protection workers interpret and operationalize vulnerability. In-depth interviews and life history calendars were collected from 44 male migrant youth who arrived in Greece as UAMs but had since transitioned into adulthood. Analysis of in-depth interviews and life history calendars examined how both cumulative disadvantage and engagement with the shelter system altered youths' trajectories into adulthood. Younger adolescents were perceived as more vulnerable and prioritized for shelters over those who were "almost 18" years old. However, a subset of youth who requested shelter at the age of 17 had experienced prolonged journeys where they spent months or years living on their own in socially isolated environments that excluded them from developmental experiences conducive to adolescent development. The shelter system for UAMs in Greece enabled youth to develop new skills and networks that facilitated integration into society, and transferred them into adult housing when they turned 18 so that they could continue developing new skills. Those who were not in shelters by the time they turned 18 could not access adult housing and lost this opportunity. Limitations included inability to capture all nationalities of UAMs in Greece, though the two most common nationalities were included.<br>Conclusions: Due to the way vulnerability was interpreted and operationalized by the shelter system for UAMs, youth who requested shelter at "almost 18" but spent extended periods of time living alone prior to arriving in Greece had the greatest need to learn new skills to facilitate their integration, but often the least opportunity to do so.

Powered by Editorial Manager® and ProduXion Manager® from Aries Systems Corporation

	Following UAMs' trajectories into early adulthood was critical in capturing this long-term consequence of the shelter system's operationalization of vulnerability.
**Suggested Reviewers:**	Bernadette Nirmal Kumar<br>Associate Professor of Global Health; Director of the Norwegian Centre for Migra, Norwegian Institute of Public Health; University of Oslo<br>BernadetteNirmal.Kumar@fhi.no<br>Expertise in migrant health in Europe.
	Miriam Orcutt<br>Senior Research Fellow in Forced Migration and Health, University College London Institute for Global Health<br>miriam.orcutt@gmail.com<br>Expertise in forced migration and health in Europe.
**Opposed Reviewers:**	
**Additional Information:**	
**Question**	**Response**
**Financial Disclosure**<br><br>Enter a financial disclosure statement that describes the sources of funding for the work included in this submission. Review the submission guidelines for detailed requirements. View published research articles from *PLOS Medicine* for specific examples.<br><br>This statement is required for submission and **will appear in the published article** if the submission is accepted. Please make sure it is accurate.<br><br>**Unfunded studies**<br>Enter: *The author(s) received no specific funding for this work.*<br><br>**Funded studies**<br>Enter a statement with the following details:<br>• Initials of the authors who received each award<br>• Grant numbers awarded to each author<br>• The full name of each funder<br>• URL of each funder website<br>• Did the sponsors or funders play any role in the study design, data collection and analysis, decision to publish, or preparation of the manuscript?<br>• **NO** - Include this sentence at the end of your statement: *The funders had no role in study design, data collection and analysis, decision to publish, or preparation of the manuscript.*<br>• **YES** - Specify the role(s) played.	This study was funded by the Johns Hopkins Center for Qualitative Studies in Health and Medicine's Dissertation Improvement Award (https://www.jhsph.edu/departments/health-behavior-and-society/research-and-centers/center-for-qualitative-studies-in-health-and-medicine/index.html) and the Pulitzer Center on Crisis Reporting (https://pulitzercenter.org). Both awards were given to author DM. Neither awards have grant numbers. The funders had no role in study design, data collection and analysis, decision to publish, or preparation of the manuscript.

* typeset	
**Competing Interests**<br><br>Use the instructions below to enter a competing interest statement for this submission. On behalf of all authors, disclose any competing interests that could be perceived to bias this work—acknowledging all financial support and any other relevant financial or non-financial competing interests.<br><br>This statement **will appear in the published article** if the submission is accepted. Please make sure it is accurate. View published research articles from *PLOS Medicine* for specific examples.<br><br>**NO authors have competing interests**<br><br>Enter: *The authors have declared that no competing interests exist.*<br><br>**Authors with competing interests**<br>Enter competing interest details beginning with this statement:<br><br>*I have read the journal's policy and the authors of this manuscript have the following competing interests: [insert competing interests here]*<br><br>* typeset	The authors have declared that no competing interests exist.
**Ethics Statement**<br><br>Enter an ethics statement for this submission. This statement is required if the study involved:<br>• Human participants<br>• Human specimens or tissue<br>• Vertebrate animals or cephalopods<br>• Vertebrate embryos or tissues<br>• Field research	This study received approval from the Johns Hopkins Bloomberg School of Health Institutional Review Board, with the approval number "IRB 8788". Oral consent was obtained from participants due to potential risk of identification from written signatures.

Write "N/A" if the submission does not require an ethics statement.<br><br>General guidance is provided below. Consult the submission guidelines for detailed instructions. **Make sure that all information entered here is included in the Methods section of the manuscript.**	

Powered by Editorial Manager® and ProduXion Manager® from Aries Systems Corporation

Format for specific study types

Human Subject Research (involving human participants and/or tissue)

- Give the name of the institutional review board or ethics committee that approved the study
- Include the approval number and/or a statement indicating approval of this research
- Indicate the form of consent obtained (written/oral) or the reason that consent was not obtained (e.g. the data were analyzed anonymously)

Animal Research (involving vertebrate animals, embryos or tissues)

- Provide the name of the Institutional Animal Care and Use Committee (IACUC) or other relevant ethics board that reviewed the study protocol, and indicate whether they approved this research or granted a formal waiver of ethical approval
- Include an approval number if one was obtained
- If the study involved *non-human primates*, add *additional details* about animal welfare and steps taken to ameliorate suffering
- If anesthesia, euthanasia, or any kind of animal sacrifice is

Powered by Editorial Manager® and ProduXion Manager® from Aries Systems Corporation

part of the study, include briefly which substances and/or methods were applied<br><br>**Field Research**<br><br>Include the following details if this study involves the collection of plant, animal, or other materials from a natural setting:<br>• Field permit number<br>• Name of the institution or relevant body that granted permission	
**Data Availability**<br><br>Authors are required to make all data underlying the findings described fully available, without restriction, and from the time of publication. PLOS allows rare exceptions to address legal and ethical concerns. See the PLOS Data Policy and FAQ for detailed information.<br><br>A Data Availability Statement describing where the data can be found is required at submission. Your answers to this question constitute the Data Availability Statement and **will be published in the article**, if accepted.<br><br>**Important:** Stating 'data available on request from the author' is not sufficient. If your data are only available upon request, select 'No' for the first question and explain your exceptional situation in the text box.<br><br>Do the authors confirm that all data underlying the findings described in their manuscript are fully available without restriction?	No - some restrictions will apply

**Describe where the data may be found in full sentences. If you are copying our sample text, replace any instances of XXX with the appropriate details.**<br><br>• If the data are **held or will be held in a public repository**, include URLs, accession numbers or DOIs. If this information will only be available after acceptance, indicate this by ticking the box below. For example: *All XXX files are available from the XXX database (accession number(s) XXX, XXX.*).<br>• If the data are all contained **within the manuscript and/or Supporting Information files**, enter the following: *All relevant data are within the manuscript and its Supporting Information files.*<br>• If neither of these applies but you are able to provide **details of access elsewhere**, with or without limitations, please do so. For example:<br><br>*Data cannot be shared publicly because of [XXX]. Data are available from the XXX Institutional Data Access / Ethics Committee (contact via XXX) for researchers who meet the criteria for access to confidential data.*<br><br>*The data underlying the results presented in the study are available from (include the name of the third party and contact information or URL).*<br>• This text is appropriate if the data are owned by a third party and authors do not have permission to share the data.<br><br>* typeset	Data cannot be shared publicly because participants are asylum seekers with ongoing legal cases that may be affected by data. Full transcripts of in-depth interviews may identify participants even after names and other standard identifying information is removed, and participants' safety may be at significant risk if they are identified. For this reason, making interview transcripts publicly available is contrary to the conditions of ethical approval from the Johns Hopkins School of Public Health Institutional Review Board. Data are available from the Johns Hopkins Center for Humanitarian Health (contact via humanithealth@jhu.edu or pbspiegel@jhu.edu) for researchers who meet the criteria for access to confidential data.
Additional data availability information:	Tick here if your circumstances are not covered by the questions above and you need the journal's help to make your data available.

Powered by Editorial Manager® and ProduXion Manager® from Aries Systems Corporation

PLOS Medicine
Call for Papers
Special Issue on Refugee and Migrant Health

October 9, 2019

Dear Editors,

I am writing to submit the manuscript *"Neither minor, but not yet adults": Interpretations of vulnerability and cumulative disadvantage for unaccompanied adolescent migrants in Greece* by Divya Mishra, PhD, Paul Spiegel, MD-MPH, Vasileia Digidiki, PhD, and Peter Winch, MD-MPH for consideration for publication in PLOS Medicine. The aforementioned manuscript is an original research article in response to the call for papers for PLOS Medicine Special Issue on Refugee and Migrant Health. A draft of this manuscript was shared with PLOS Acting Editor-in-Chief Clare Stone, who stated that the topic is within scope and encouraged submission for the special edition.

This study addresses how child protection organizations in Greece interpret some unaccompanied minors as more vulnerable than others when prioritizing them for specialized children's shelters, and examines how this operationalization of vulnerability addresses the psychosocial needs that these minors have when they arrive in Greece. The study analyzes the life trajectories of former unaccompanied minors and is therefore able to capture how placement in children's shelters impacts unaccompanied youths' early adulthood. We believe this study is suitable for the special edition in that it addresses the health of migrants as they move through low-, middle-, and high-income countries. This research will inspire researchers and clinicians who work with refugees and migrants to take a more holistic perspective of unaccompanied minors' needs, and to consider how organizational practices may have long-term impacts on these minors' lives.

We declare that this manuscript is original, has not been published before and is not currently being considered for publication elsewhere.

We know of no conflicts of interest associated with this publication, and there has been no significant financial support for this work that could have influenced its outcome. As Corresponding Author, I confirm that the manuscript has been read and approved for submission by all the named authors.

We hope you find our manuscript suitable for publication and look forward to hearing from you in due course.

Sincerely,

Divya Mishra
Department of International Health, Johns Hopkins School of Public Health
Tel: +13023832265
Email: divya.mishra@jhu.edu; divya.mishra.med@dartmouth.edu

Manuscript
Click here to download Manuscript Neither minor, but not yet adults_PLOS Medicine Submission.docx

Full title: "Neither minor, but not yet adults": Interpretations of vulnerability and cumulative disadvantage for unaccompanied adolescent migrants in Greece

Short title: Vulnerability and cumulative disadvantage of unaccompanied minors in Greece

Divya Mishra[1]

Paul B. Spiegel[1,2]

Vasileia L. Digidiki[3]

Peter J. Winch[1]

1. Department of International Health, Johns Hopkins Bloomberg School of Public Health, Baltimore, MD, USA
2. Center for Humanitarian Health, Johns Hopkins Bloomberg School of Public Health, Baltimore, MD, USA
3. François-Xavier Bagnoud Center for Health and Human Rights, Harvard School of Public Health, Boston, MA, USA

All affiliations are current.

Corresponding author:

Divya Mishra, divya.mishra@jhu.edu

Abstract

Background: In settings of mass displacement, unaccompanied minors (UAMs) are recognized as a vulnerable group and consequently prioritized by relief efforts. This study examines how the operationalization of vulnerability by the national shelter system for male UAMs in Greece shapes their trajectories into adulthood.

Methods and Findings: In 2018-2019, key informant interviews were carried out in organizations that refer UAMs to specialized shelters in Athens to understand how child protection workers interpret and operationalize vulnerability. In-depth interviews and life history calendars were collected from 44 male migrant youth who arrived in Greece as UAMs but had since transitioned into adulthood. Analysis of in-depth interviews and life history calendars examined how both cumulative disadvantage and engagement with the shelter system altered youths' trajectories into adulthood.

Younger adolescents were perceived as more vulnerable and prioritized for shelters over those who were "almost 18" years old. However, a subset of youth who requested shelter at the age of 17 had experienced prolonged journeys where they spent months or years living on their own in socially isolated environments that excluded them from developmental experiences conducive to adolescent development. The shelter system for UAMs in Greece enabled youth to develop new skills and networks that facilitated integration into society, and transferred them into adult housing when they turned 18 so that they could continue developing new skills. Those who were not in shelters by the time they turned 18 could not access adult housing and lost this opportunity. Limitations included inability to capture all nationalities of UAMs in Greece, though the two most common nationalities were included.

Conclusions: Due to the way vulnerability was interpreted and operationalized by the shelter system for UAMs, youth who requested shelter at "almost 18" but spent extended periods of time living alone prior to arriving in Greece had the greatest need to learn new skills to facilitate their integration, but often the least opportunity to do so. Following UAMs' trajectories into early adulthood was critical in capturing this long-term consequence of the shelter system's operationalization of vulnerability.

Introduction

The number of migrant children traveling unaccompanied has been rising globally over the previous decade (1-3). In Europe, the number of unaccompanied minors (UAMs) spiked from 13,800 in 2013 to 23,300 in 2014, then to an unprecedented 96,000 in 2015. Actual numbers are likely higher, as many UAMs remain undocumented by authorities (4). In Greece, UAMs made up an estimated 35% of the migrants who arrived in 201[illegible] 6). Restrictive immigration policies implemented by the European Union in the years that followed have transformed Greece into a de facto buffer zone for migrant[illegible], where backlogged asylum and family reunification processes keep UAMs in limbo for years (8). At the time of this writing, Greece hosts estimated 32,000 migrant children, 4,000 of whom are registered as unaccompanied or separated (9).

UAMs are considered an especially vulnerable subgroup of migrants in humanitarian contexts and consequently prioritized for aid (10). Vuln[illegible]bility, according to the International Organization of Migration (IOM), is “the diminished capacity of an individual or group to have their rights respected, or to cope with, resist or recover from exploitation, or abuse” (11). In order to protect UAMs, both local and international NGOs in Greece operate shelters for unaccompanied children. However, these shelters are scarce, and approximately two-thirds of UAMs who requested shelter in 2018 and 2019 were waitlisted every month (12, 13). Over 90% of UAMs in Greece, as in most of Europe, are males between 15-17 years of age (13-15). Consequently, they are only eligible for shelters during the brief time before they turn 18 and are categorized as men, at which point they are no longer seen as vulnerable or prioritized for aid (16-18).

Empirical research has found that, contrary to widespread assumptions regarding young men’s vulnerability or lack thereof, male gender and traveling unaccompanied are statistically significant indicators of vulnerability to exploitation among minor and adult migrants (11). No significant difference

in indicators of exploitation was found between UAMs and adult males until the age of 27 (11). Substantiating these findings, a study by Care International found that unaccompanied single males, adults and minors alike, commonly experienced sexual and economic exploitation in Greece and did not receive institutional support necessary to exit exploitative circumstances (15). The continued exploitation of unaccompanied young males even in adulthood may be explained by environmental factors that remain unchanged as youth transition from adolescence into adulthood.

The trajectories that bring UAMs to Europe are sometimes circuitous, encompassing various legal statuses, living conditions, and changing motivations (19), with long periods of immobility punctuated by bursts of mobility (19-21). Many make multiple attempts to arrive at their destinations, while others move on from intended "destination" countries when faced with hardship or unexpected opportunities (22). Prolonged journeys, lasting longer than 3 months, are associated with traveling unaccompanied and male gender, as well as a higher incidence of exploitation (4, 11). Approximately 25% of UAMs entering Greece experienced journeys lasting 3-6 months, while 13% experienced journeys longer than 6 months (4). UAMs' experiences during these months remain largely unknown.

Unlike most adolescents, UAMs come of age away from families and communities that can structure their transition to adulthood, such as rites of passage (23-25), and experiences that help form adult identities (26, 27). Nonetheless, the different social environments they inhabit (28) influence their physical, psychosocial, and emotional development (29, 30). To understand how these social environments shape UAMs' progression to adulthood, this study draws on life course theory (31, 32), which examines transitions that mark adolescents' life trajectories, such as leaving childhood homes, changing legal statuses, or moving to a new country, some of which function as turning points and alter youths' life trajectories.

Life course theory highlights how trajectories of disadvantaged youth are shaped by early life circumstances (33). Cycles of reciprocal interactions between an individual's reaction to his disadvantage, and the environment's response to his reaction cause disadvantage to accumulate over years and lead to poor outcomes in adulthood. Adolescents' isolation from society in particular has been identified as an important disadvantage with repercussions in adulthood (33, 34). Using life course theory, this study examines UAMs' concurrent developmental and migratory trajectories to understand how they become exposed to exploitative circumstances, as well as how the operationalization of vulnerability by Greece's shelter system impacts their trajectories into adulthood.

Methods

We conducted interviews in Athens in 2018-2019 from NGO staff and migrant youth. In order to protect the identities of study participants, names of NGOs, child protection workers, and migrant youth were replaced with pseudonyms. Key informant interviews were carried out with child protection workers at NGO X, NGO Y, and Transitional Shelter Z for young adults. NGO X referred homeless and unstably housed UAMs to shelters and provided holistic case management services until the youth were successfully placed. Two lawyers, a social worker, a psychologist, and a cultural mediator were interviewed at NGO X. NGO Y operated one specialized shelter for UAMs, transferred UAMs from the islands to the mainland, provided legal guardians for UAMs, and referred them to other shelters when needed. A social worker and project manager were interviewed at NGO Y. Transitional Shelter Z was staffed by a social worker and psychologist. It housed 24 young adult males who had arrived in Greece as UAMs. Interviews focused on processes through which youth were placed in shelters. A total of 9 staff members from these three organizations were interviewed. Interview transcripts were qualitatively analyzed using open, inductive coding. Codes focused on how NGO staff interpreted and addressed UAMs' vulnerability in their day-to-day work.

To understand UAMs' life trajectories, in-depth, semi-structured interviews were conducted with 44 male migrant youth, aged 18-21, who arrived in Greece as UAMs. Interviews allowed participants to construct a narrative of the events surrounding their departure from home countries up through the time of the interview. In order to maximize the diversity among participants, initial ethnographic assessment in the form of participant-observation at food distribution sites for the homeless and a youth center for 16- to 21-year-old migrants was used to identify different subgroups of migrant youth, such as those in shelters, and those who were homeless or squatting in abandoned buildings. Then, snowball sampling was used starting with seeds in each identified subgroup (35). At the time they were interviewed, 16 youth were either homeless or informally housed, and 28 were living in NGO-provided accommodation facilities. Interviews were conducted in Farsi, Dari or Urdu, as the two most common nationalities among UAMs in Greece are Afghan and Pakistani (11). Female UAMs were excluded because they make up less than 10% of UAMs in Europe (14, 15) and would require alternative recruitment strategies. Oral consent to study procedures was obtained from participants at the start of each interview.

Alongside each interview with migrant youth, a semi-structured life history calendar (36) was constructed to capture the trajectories that participants' lives had taken. The life history calendar took the form of a timeline that identified changes in participants' living situations, how long each living situation lasted, and sources of support that were available in each situation. This method was able to capture how participants' circumstances changed as they moved between countries and within countries, such as from one accommodation facility to another. Due to the open-ended nature of interviews, some participants provided information regarding their life history prior to their departing their home countries while others did not. To have comparable data on life history prior to departure for all participants, more structured follow-up interviews were conducted to extend life history calendars to cover the period between birth and departure from home country, with a focus on 1) place and type of

residence during each year of life, 2) sources of economic support in each place of residence, 3) co-inhabitants in each residence, 4) years of schooling, and 5) experience with independent decision making. Only 32 of 44 participants could be reached for follow-up interviews.

To analyze life history calendars and interview transcripts, participants' lives were divided into 4 different time periods. These were 1) from birth to departure from home country, 2) from departure to arrival in Greece, 3) as UAMs in Greece, and 4) from age 18 to the time of interview. Challenges, forms of support, and access to resources were identified in each time period, and their impact on later periods of life were qualitatively assessed. To understand how placement in children's shelters impacted life trajectories, the life history calendars of youth who were and were not placed in children's shelters were compared. This study was approved by the Johns Hopkins School of Public Health Institutional Review Board.

Results

Determination of vulnerability

To place a homeless or informally housed UAM in a children's shelter, child protection organizations sent a referral on his behalf to EKKA, the Greek National Center for Social Solidarity. To secure placement typically took several months, unless the UAM in question was considered exceptionally vulnerable, in which case, placement was often arranged within a month. Social worker R. and psychologist M. from NGO X explained that EKKA's interpretation of UAMs' vulnerability differed from their own interpretations.

R: EKKA does not have the opportunity to meet each unaccompanied minor for whom they receive referral for accommodations. So that means they don't…exactly understand the situation of the minor.

M: If EKKA [is looking at] five cases, and they're all from Pakistan, 16-years-old, maybe hosted by some friends now and then, [and] they have no papers—they have the same criteria. How will [EKKA] prioritize? Of course, if they see someone 15, or 14, they prioritize them…But sometimes, we see that someone 15-years-old feels safe where he is hosted. We can also see someone who is 16 or 16-and-a-half who doesn't give the impression that he feels safe… If the child is dirty, [or] if he is totally homeless [and] it seems as if the child has not taken a bath in ten days… [Or] if he has not had food to eat for five days…[Then] we decide to prioritize his case.

Social worker R. explained that, though it was possible to emphasize factors other than age that were indicative of UAMs' vulnerability, whether or not this was done depended on the staff member who completed the referral:

R: We have a specific form from EKKA…The end of the template [has space for] the social history…It is in the [case] worker's role to understand what are the vulnerabilities of each minor…so that they can write them down in the social history, and EKKA can be informed about why that child is more vulnerable than the other, for example…[But] it depends on the case worker.

M: [The case worker] may write one or two sentences, that [the UAM] is homeless, that he is distressed. [This] vague, general information doesn't help the boy.

Though key informants reported that descriptions of factors of vulnerability other than age can counter the assumption that the youngest UAMs are the most vulnerable, their ability to do so seemed to diminish as UAMs approached adulthood. Psychologist M. highlighted the challenges faced by homeless and informally housed UAMs as they approached their 18th birthday:

> M: The problem is, there is a prioritization of the younger ages…If they are almost 18, it is a very gray zone, because they are neither minor—let's say, not very, very vulnerable, though of course, it is not just about their age—but they are not adults yet. We try to help them, but it is very difficult for an almost-18-year-old to enter a shelter.

Concurring with Psychologist M., Social worker V. of NGO Y stated:

> V: You can understand that, someone who is 16 years old can be easily prioritized to be placed in the facility. Someone who is two months until 18 would not be that eligible. I mean, they are eligible under what the law says. But they will not be prioritized.

Cumulative disadvantages

In order to illustrate how cumulative disadvantages shaped UAMs' trajectories and how placement in children's shelters can alter them, the following sections follow the experiences of three youths whose experiences were repre[illegible]tative of the 44 study participants. These young men are Gauhar (Afghan, 19-years-old, arrived via the land route at age 17), Hafez (Iranian, 18-years-old, arrived on Samos island at age 16), and Bilal (Pakistani, 19-years-old, arrived via land route at age 17). Table 2 presents their experiences prior to leaving their home countries, Table 4 presents their experiences while traveling unaccompanied, and Table 5 presents their experiences in Greece.

Disadvantages prior to departure

The disadvantages that youth faced in their home countries shaped the experiences they had along their journeys. UAMs whose families were struggling financially often could not pay a smuggler to take them all the way to Greece. These youth spent months or years in Iran or Turkey homeless or working in exploitative conditions. Youth whose families had extensive connections with diasporic communities were often able to seek help from a relative or family acquaintance even though they were unaccompanied. Table 1 describes how disadvantages that youth experienced prior to their departure shaped future experiences, often leading to further disadvantages. This table is not intended to provide an exhaustive list, but rather to illustrate the impact that early disadvantages can have.

Table 1. Disadvantages prior to departure

Disadvantage	Explanation	Illustrative quote
History of displacement	Youth who were displaced with their families prior to traveling unaccompanied often grew up in circumstances where they had limited rights, and their families had few assets.	"I am Afghan, but I was born in Iran…Afghan refugees are not allowed to go to school in Iran so I came to Turkey to study. But, I couldn't study in Turkey because I had to work to make a living." —Jamal, Afghan, 19-years-old, arrived on Lesbos island at age 17
Death or disappearance of parent(s)	Youth who lost one or both parents as children were susceptible to neglect, poverty, limited educational opportunities, and child labor. The death	"When my father passed away, we had no breadwinner in the family to take care of us in Kabul. My aunts and uncles were in Pakistan...so we decided to move there…I

	of fathers in particular caused economic hardship.	had to drop out of school, because I had studied up to the eight grade in Afghanistan…If I wanted to study in Pakistan, I would have to start from the first grade." —Mahdi, Afghan, 19-years-old, arrived on Lesbos island at age 17
Lack of education	Youth who had limited access to education usually could not communicate in English when they arrived in Greece. Some were not literate in any language. Youth who did not spend time in school also had less experience interacting with adults in institutionalized settings.	"When I came to Lesbos, they taught us how to read and write [English], and I learned a little bit…I cannot read or write in my first language. If someone messages me [in Dari] on Facebook, I can't write back." —Masood, Afghan, 19-years-old, arrived on Lesbos island at age 17
Ability to pay smuggler	Youth whose families could only afford to pay a smuggler to take them as far as Iran or Turkey, instead of all the way to Greece, often spent long periods of time homeless or working undocumented before they reached Greece.	"We had only arranged with the smuggler to take me as far as Turkey…My mother paid…She sold her jewelry…and then I stayed and worked…I met some other Afghans, and I found [factory] work through them." —Fayaz, Afghan, 18-years-old, arrived on Lesbos island at age 14

Contacts in diaspora communities	Youth whose families had contacts within diaspora communities sought help from these contacts to avoid homelessness and access other information and resources when they were unaccompanied.	"I didn't have anywhere decent to live…I didn't have any relatives in Greece…Then I called home and asked if there was anyone from our village here…In two, three days, I found someone…I went and lived with him for a month." —Hasib, Bangladeshi, 19-years-old, arrived on Lesbos island at age 17

Gauhar and Hafez, as described in Table 2, experienced many of these disadvantages prior to their arrival. Gauhar's family was displaced from Afghanistan to Pakistan, where he was born. They family did not own property, and Gauhar never went to school. Though Gauhar's father was still alive, he became too ill to work, at which point Gauhar, aged 10, started working to support the family. Hafez lost both of his parents by the time he was 9-years-old, at which point he dropped out of school. He lived with relatives and family friends for short periods of time, and by age 12, had started working in construction. At age 14, he rented an apartment with his little brother. Both Gauhar and Hafez could only afford to pay a smuggler to take them as far as Turkey.

Bilal had several advantages prior to his departure (see Table 2). Though his parents could not afford to send him to university in Pakistan, they lived on their own property, and could afford to pay a smuggler to take Bilal directly to Europe. Bilal had also finished high school before he left and was fluent in English.

Table 2. Disadvantages prior to departure

Gauhar *(Afghan, 19-years-old, arrived via land route at age 17)*

Gauhar was born in Peshawar, Pakistan, where his family were registered refugees. He lived with his mother, father, and four siblings in a dirt house that his father rented from the money he earned as a taxi driver. Gauhar never went to school.

When Gauhar was 10 years old, his father was diagnosed with cancer and could no longer work. The family returned to Afghanistan and rented a house in rural Langarhar, where his older brother began working as a rickshaw driver and Gauhar worked as a part-time butcher's assistant to support the family and pay for their father's medical bills. When Gauhar was 13-years-old, local Taliban attempted to recruit him, and his

Hafez *(Iranian, 18-years-old, arrived on Samos island at age 16)*

Hafez was born in Mashhad, Iran. His father passed away when he was 2-years-old, at which time his mother moved him and his younger brother to an apartment in the town of Gonbad, where she worked as a teacher. When Hafez was 9 years old, his mother, too, passed away. Hafez stopped attending school after his mother died.

Hafez and his brother spent 3 years living with their aunt, who Hafez found intolerably harsh. When he was 12 and his brother 11, they left their aunt's house and were briefly homeless. However, a man who knew their mother offered to let them stay in a room above his garage in exchange for their assistance at his mechanic's shop.

Bilal *(Pakistani, 19-years-old, arrived via land route at age 17)*

Bilal was born in Peshawar, Pakistan into a large, joint family household. His mother came from an educated family of lawyers, whereas his father's side of the was implicated in local gang violence. When Bilal was 14 years old, his parents sent him to a English-medium boarding school in a different town, away from the violence that the family was embroiled in. At the age of 17, Bilal's parents told him they couldn't afford to send him to university. After a family discussion, Bilal and his parents decided to send him to Europe, in hopes that he could build a life, and maybe even continue his education in a place safe from violence. With money borrowed

mother told him to leave Afghanistan. His brother paid a smuggler 1200 USD to take him to Turkey.	Hafez worked at a construction site while his brother assisted the mechanic. When Hafez was 14-years-old, he and his brother rented an apartment of their own. An elderly Christian woman who lived nearby helped the boys frequently—"she became like my mother," Hafez said—and Hafez decided to convert to Christianity, which constituted a crime in Iran. When some relatives threatened to report him to authorities, an aunt paid a smuggler to take him to Turkey where he could avoid persecution. At the time, he was 16-years-old.	from relatives, Bilal's family paid a smuggler to take him to Italy.

Disadvantages as an irregular child migrant

Once youth left their home countries, their experiences fell into two broad categories: being transported by smugglers and living alone. The experience of being transported by smugglers was often dangerous and traumatic, as described by Fayaz (Afghan, 18-years-old, arrived on Lesbos island at age 14).

> F: I started my journey from Nimroz (Afghanistan)…Along the route, there were mountains, [and] a desert. Sometimes, we walked on foot for one whole day and night, for 24 hours. Sometimes, [they] took us in a car, up to 12 or 16 people in a car the size of a normal taxi. They even put people in the trunk.
>
> My worst memories are of the car, [with] 12 or 16 of us in a car. They mistreated us, and we couldn't do anything because it wasn't our country. The agents (smugglers) harassed the Pakistanis a lot, violently. They harassed Afghans, too, yanking our hair, and things like that. For someone who hasn't seen all this, he becomes mentally unhinged when he sees it for the first time.

While in the process of being transported, youth were not necessarily on the move every day. When there was high police activity, or if weather conditions were harsh, migrants were kept waiting for weeks, even months, in smugglers' safe houses, called *musafer khanas* in both Urdu and Dari/Farsi. However, the intention of further travel was always present.

There were few independent decisions that youth needed to make—or even could make—while being transported by smugglers. The food they ate, the amount of water they drank, where they stayed and how they traveled, were all determined by smugglers and their associates. The following excerpt from Mohammad (Bangladeshi, 19-years-old, arrived on Lesbos island at age 17) demonstrates the lack of autonomy youth experienced when traveling with smugglers.

> M: One day, I tried to tell [the agent] that I had a fever and couldn't walk. It was cold and raining really hard, and we had to walk outside. When I told the agent, he started to beat me. I was like, "Why are you doing this? I have a fever, and you are beating me?"

> He said, “You will have to walk. If you stay here, the police will catch you. If the police catch you, and they ask you how you got here, you will tell them about me…I'm not going to get caught for you. So you walk. If you die, I will toss your body to the side of the road.”

In contrast to when they were being transported, once youth were taken to an agreed upon destination and accounts with smugglers were settled, they were left on their own. This was many UAMs’ first experience making decisions without adult supervision. Table 3 gives an overview of the types of circumstances that migrant youth found themselves in while living on their own. Common among these varying circumstances was the fact that youth were socially isolated, without a peer group or supportive community, and excluded from activities that would help them build new networks or skills.

Table 3. Circumstances experienced while living alone

Circumstances	Explanation	Illustrative quote
Homeless	While they were homeless, youth had little to no social support. They could not participate in activities or build relationships that might prepare them to be self-sufficient adults, and their desperation to meet basic needs, like food and shelter, left them vulnerable to exploitation.	“When I came to Turkey, I slept on the streets…After a month and a half, a Turkish man asked me why I sleep outdoors. I told him I didn’t have a place to stay and he took me to his house…Two days later, he asked me to work with him on a construction site. I worked with him, but he didn’t mention anything about paying me.” —Asgar, Afghan, 18-years-old, arrived on Lesbos island at age 16

Working	Youth who worked while living abroad typically worked in exploitative industries where they were vulnerable to injuries, paid very little, and denied opportunities for personal growth and development as adolescents. Some were loosely supervised by relatives, while others were on their own.	"I lived in Turkey for about a year…It was very difficult work. I worked 13 or 14 hours a day in a factory that made and packaged speakers…I was just so tired afterwards. I really like football, but I could only watch it on TV, I couldn't play. I was fed up."<br>—Fayaz, Afghan, 18-years-old, arrived on Lesbos island at age 14
In the care of relatives	Even in the care of relatives they trusted, youths' participation in host societies was limited due to their undocumented status, or differences race, religion, or language. They remained isolated from their peers.	"I was doing well in Iran…My uncle took care of me…I didn't meet the people of Iran, because I am Sunni and they are Shia. My uncle said, stay home, but if you want to go out…don't talk to [Iranians], and don't pray outside…If they see you pray [like a Sunni], they might attack you."<br>—Mohammad, Bangladeshi, 19-years-old, arrived on Lesbos island at age 17
Passing time	A minority of youth were able to request enough money from their families back home, through agencies like Western Union or MoneyGram, and did not need to work while in Iran or Turkey. However, these youth were still undocumented and	"There was nothing for me to do [in Turkey]. I wasn't in a good place…I just wandered about, from place to place, to internet cafés…I used to call home for money whenever I needed anything."

	unable to participate or integrate into host societies.	—Tariq, Pakistani, 19-years-old, arrived directly at Greek mainland at age 17

UAMs whose families could afford to pay smugglers to take them all the way to Greece often did not experience living alone at all. Bilal (see Table 4) was transported by smugglers for the entire duration of his one-month journey from Pakistan to Greece. Furthermore, his father gave him additional cash to pay smugglers for better treatment.

Gauhar and Hafez (Table 2) had only paid to be taken as far as Turkey. Gauhar was in Turkey for 4 years, during which he was homeless, then later paid rent to stay in someone's basement. He collected cardboard scraps to earn money, most of which he spent on rent. It was only when he felt threatened by a local trafficking gang that he paid a smuggler to take him to Greece. Hafez spent 6 months working in a clothing factory in Turkey before he unexpectedly received an opportunity to go to Greece.

Table 4. Experiences in transit

Gauhar *(Afghan, 19-years-old, arrived via land route at age 17)*	Hafez *(Iranian, 18-years-old, arrived on Samos island at age 16)*	Bilal *(Pakistani, 19-years-old, arrived via land route at age 17)*
When Gauhar arrived in Turkey, he was undocumented, had nowhere to go, and slept in a park. While he was homeless, he saw other migrants collecting cardboard boxes from the streets and trash bins. He asked these other	When he crossed the border into Turkey, Hafez boarded a bus to Istanbul, where he went to a neighborhood nicknamed Iranian street to look for a job. He found work in a factory making jeans and paid rent to stay in an apartment	Bilal recalled his journey to Europe as terrifying. He witnessed Iranian border security shoot at migrants, was made to walk for entire days without water, ordered to run through the night by the smugglers. He was slapped by a

migrants and discovered that he could get paid for collecting cardboard. He began collecting and selling cardboard as well, and earned about 800 Lira (130 USD) per month). Not long after he had been doing this work, a Kurdish man saw him sleeping in the park and offered him a basement room for 500 Liras per month. For four years, Gauhar spent his days collecting cardboard and sleeping in the Kurdish man's basement.<br><br>When Gauhar was 17 years of age, he got into a knife fight with a local trafficking gang when they wanted to sell a homeless woman he was close to. From that point on, he continued to face threats of violence from the gang and decided to leave Turkey in search of safety, using he savings to pay a smuggler to take him to Greece.	with 4 other Iranians. He had been working in the factory for 6 months when his smuggler contacted him saying that his aunt paid him to take Hafez to Greece. Hafez was still 16-years-old when he left for Greece.	smuggler once for lagging behind the group. However, he said journey was often safer than that of his co-travelers. Prior to his departure, Bilal's father had equipped him with US dollars, the dominant currency in Asia's smuggling networks, and instructed him to tip the guides and drivers who transported him to avoid harassment. By tipping preemptively, Bilal believed he was usually able to secure relatively comfortable arrangements, even as he watched other travelers get forced into the trunk of a car by smugglers. It took Bilal approximately a month to arrive in Greece, during which he was continuously transported by smugglers.

The role of shelters for unaccompanied minors

After their arrival in Greece, Hafez and Bilal were eventually, though not immediately, placed in shelters for UAMs (Table 5). Shelters facilitated access to Greek lessons, and both young men were fluent in Greek at the time they were interviewed. Bilal was able to resume his education and had plans to apply for university. Hafez did not pursue higher education but had plans to participate in the Greek economy by opening up a fruit stand. Both Hafez and Bilal were transferred to adult accommodations after they turned 18, where they were able to continue learning Greek, attend school, and pursue activities that facilitated their integration into Greek society. Since they lived in NGO provided housing, they did not have to pay rent. They also received cash assistance from UNHCR to help cover their basic needs.

Despite having requested accommodations while he was a minor, Gauhar was not placed in a shelter before he turned 18. Since he was homeless at 18, he could not to apply for the cash assistance that most adult asylum seekers received, as the application required a mailing address. Unable to find other work, Gauhar sold sex in order to survive, and considered trying to go to jail in order to sleep indoors.

Psychologist M. from NGO X explained that, if, like Gauhar, homeless or informally housed UAMs were not placed in children's shelters before they turned 18, it was unlikely that they would receive any kind of housing assistance at all.

> M: More than 18, and [the youth] is considered a single man…Single men are not very prioritized for accommodations, whether it is a shelter, even if it is a camp, [or] it is the apartments [provided] by UNHCR…So, it is very difficult when [UAMs] are almost 18.

Supporting M.'s comments, the manager of Transitional Shelter Z revealed that 16 of the 24 young adults who lived there had been transferred from children's shelters. The remaining 8 had been

transferred from camps, suggesting that it was unlikely for youth like Gauhar, who were not placed in any kind of NGO-provided accommodations before turning 18, to receive accommodations in adulthood.

Table 5. Comparative case studies, part 3: Experiences in Greece

Gauhar *(Afghan, 19-years-old, arrived via land route at age 17)*	Hafez *(Iranian, 18-years-old, arrived on Samos island at age 16)*	Bilal *(Pakistani, 19-years-old, arrived via land route at age 17)*
Upon arriving in Greece, Gauhar was intercepted by police and detained for one month. After he was released, he worked at a construction site for 3 days and made €100. He then bought a train ticket to Athens, where he registered for asylum. In Athens, he requested accommodations but turned 18 before he was placed in a shelter. At the time he was interviewed, Gauhar had been homeless in Victoria Square for approximately 8 months. He not been able to find a job, and without an address, he could not apply for the monthly €150 cash assistance that adult refugees received from UNHCR. For	As soon as Hafez reached Samos island, he was taken to a nearby reception center. The reception center did not permit migrants to leave the center for a period of 20 days, at which point they were given their asylum applicant cards and were allowed to move freely within the island, but not to the mainland. Before Hafez's 20 days were up, a group of Iranians invited him to join them as they attempted to stow away on a cargo ship headed for the mainland. Hafez successfully made it to Athens, but was undocumented.<br><br>Without anywhere to go in Athens, Hafez was taken in by a group of older Iranians in Elefsina camp in	Upon arriving in Greece, Bilal realized he had been duped by his smuggler and would not be taken to Italy after all. He had nowhere to go when he arrived in Athens, and was homeless in Victoria Square for one week, and informally hosted by other migrants for 1.5 months, and given temporary accommodations by a local NGO for 2 weeks. During that time, Adil advocated to be placed in a shelter with the assistance of several NGO staff (see Chapter 2 for details). Four months before he turned 18, Adil was placed in the children's section of a camp. Six days after his 18th birthday, he was transferred to an apartment for young adults,

approximately 4 of those 8 months, Gauhar reported selling sex to Greek men for €10-20 per customer. He said he was tired of living in Victoria Square, and was considering selling drugs or getting into a fight so that the police would take him to jail, where he would have a roof over his head.	exchange for household work. This arrangement lasted 3 months, during which Hafez was abused and beaten, but kept a low profile due to his undocumented status.<br><br>Eventually, Hafez was discovered by a social worker who facilitated his asylum application and transferred to a shelter for UAMs. At the shelter, Hafez attended Greek classes, and was fluent at the time he was interviewed. When he turned 18, he was transferred from the shelter to an apartment for adults, where he could continue taking language classes without worrying about rent payments. He also received €150 a month in cash assistance from UNHCR. In 2019, he was making plans to open up a small fruit stand in Athens.	where he had been living for a year at the time he was interviewed.<br><br>Since he was placed in the camp, Adil had taken Greek lessons and repeated the 11th grade in Greece. He was looking for a summer job when he was interviewed, and had plans to repeat the 12th grade in Greece as well, with plans to eventually study engineering in university. As an adult, he received €150 a month in cash assistance from UNHCR.

Discussion

Data collected from key informant interviews suggested that there was a widespread tendency to see younger UAMs as the most vulnerable and prioritize[illegible]m for aid. Staff from NGO X acknowledged that

age was not the only determinant of UAMs' vulnerability, and that other factors, such as whether or not the UAM felt safe where he was staying, or if he wasn't able to afford food or stay clean, may be even more indicative of vulnerability than age. However, the communication of other, more subjective factors was dependent on the individual staff members who sent referral forms to EKKA. If these subjective factors were not described convincingly enough, EKKA defaulted to using UAMs' chronological age as a proxy for vulnerability. The ability of other factors to counter the perceived inverse relationship between age and vulnerability diminished after UAMs turned 17, at which point NGO staff described them as "not that eligible" or "neither minor…but not yet adult."

NGO staff's association of vulnerability with younger ages did not take into consideration the cumulative disadvantages (33) many of them had accrued by the time they requested assistance. Youth from poorer families often could not pay a smuggler to take them all the way to Europe, and therefore spent months or years either homeless or working as child labor, exposed to traumatic or exploitative conditions in environments not conducive to adolescent development. Cultural and geographic distance weakened their ties with families back home (2), and their undocumented status, along with cultural and linguistic differences, prevented them from integrating with peer groups in host countries. Living alone in such detrimental, isolated circumstances was a disadvantage that precipitated future disadvantages; the longer UAMs were living alone before arriving in Greece, the longer they were denied opportunities to build skills and networks that would help them become self-sufficient adults and integrate into Greek society. Youth who had fewer disadvantages, like Bilal, experienced fewer and shorter disruptions in their adolescent trajectories, avoided many exploitative circumstances due to financial support from their families, and had more skills that facilitated integration in Europe.

Once UAMs arrived in Greece, placement in a children's shelter constituted a turning point (32) that had the potential to alter the trajectories of lives even into adulthood. This is illustrated the trajectory of

Hafez, who, prior arriving in Greece, shared many of the disadvantages that Gauhar had. However, after he, like Bilal, was placed in a children's shelter, he received many more opportunities to integrate into Greek society than Gauhar. At the time they were interviewed, both Hafez and Bilal lived in NGO provided housing and participated in some kind of skill building activity. In contrast, Gauhar remained homeless, exposed to exploitative circumstances.

Shelters provided for UAMs' basic needs, but also gave them opportunities to network with peers their own age who had similar backgrounds and experiences. In addition, shelters connected youth to activities that could help them build new skills that would facilitate their integration in Greece. These included Greek and English language classes, vocational training, or in some cases, guidance on applying to higher education. Importantly, when these youth turned 18, they were transferred to adult accommodations where they could continue investing time in personal development, learning to participate in Greek society without worrying about basic needs like shelter.

On the other hand, UAMs who could not be placed in a shelter before they turned 18 were typically not able to access any kind of accommodation facility at all as young adult males, as they were considered no longer vulnerable. Such young men continued being homeless or relying on underground economies for survival, which exposed them exploitative circumstances, irrespective of whether they were minors or adults. In fact, as Gauhar's trajectory illustrates, turning 18 did not make these youth any less vulnerable. Their environment did not change, except the possibility of receiving aid diminished even further. The continued lack of stable accommodations further added to youths' cumulative disadvantage, as the lack of an address prevented them from applying to forms of assistance that they would have otherwise been eligible for as adults. The cumulative disadvantage that characterized these youths' trajectories constituted a kind of vulnerability on its own, as it diminished unaccompanied youths' ability to protect themselves from abuse and exploitation.

The use of semi-structured lif[illegible]story calendars (36) allowed for examination of how cumulative disability shaped UAMs' trajectories as migrants as well as developing adolescents (32, 33, 37). Furthermore, by extending youths' life history calendars into early adulthood, this study captured long-term effects of children's shelters. While some longitudinal studies incidentally capture UAMs who age out of services for minors (38, 39), most studies focus on these youth only while they remain underage, and are therefore unable to capture long-term effects of interventions.

Limitations of the study included our inability to reach all youth for follow-up interviews. Some youth had already moved out of Athens to find work by the time they were contacted, while others left Greece altogether. Some could not be reached at all. The study also did not capture all nationalities of UAMs in Greece, such as those from sub-Saharan Africa or the Middle East. However, the two most common nationalities among UAMs, Afghan and Pakistani, were well represented.

In order to avoid creating long-lasting disparities between youth who are placed in shelters and those who are not, pathways should be developed to allow young adult males to enter accommodation facilities and build skills and networks that enable them to integrate into Gr[illegible] society. Furthermore, cumulative disadvantages (33) should be taken into account while assessing UAMs' vulnerability. Youth from disadvantaged backgrounds may have more unmet needs than those with fewer disadvantages, even if they are older in age. The assumption that, as they get closer to becoming adult males, these youth are less vulnerable and therefore less in need of assistance (16, 18, 40) may inadvertently increase their exposure to exploitation.

References

1. UNICEF. A Child is a Child: Protecting Children on the Move from Violence, Abuse, and Exploitation. United Nations International Children's Emergency Fund (UNICEF); 2017. [Available from: https://www.unicef.org/publications/index_95956.html]

2. UNICEF. Uprooted: The growing crisis for refugee and migrant children. United Nations International Children's Emergency Fund (UNICEF); 2016. [Available from: https://www.unicef.org/publications/index_92710.html]

3. Bhabha J. Arendt's Children: Do Today's Migrant Children Have a Right to Have Rights? Human Rights Quarterly. 2009;31(2):410-51.

4. UNICEF. Harrowing Journeys: Children and Youth on the Move Across the Mediterranean Sea, at Risk of Trafficking and Exploitation. United Nations International Children's Emergency Fund (UNICEF); 2017. [Available from: https://www.unicef.org/publications/index_100621.html]

5. Fili A, Xythali V. The continuum of neglect: Unaccompanied minors in Greece. Social Work and Society International Online Journal. 2017;15(2).

6. IFRC. Revised Emergency Plan of Action Greece: Population Movement. International Federation of the Red Cross and Red Crescent Societies (IFRC); 2017. [Available from: adore.ifrc.org/Download.aspx?FileId=157931]

7. Zaragoza-Cristiani J. Containing the Refugee Crisis: How the EU Turned the Balkans and Turkey into an EU Borderland. The International Spectator. 2017;52(4):59-75.

8. Tondo L. Nearly 900,000 asylum seekers living in limbo in EU, figures show. The Guardian; 2019. [Available from: https://www.theguardian.com/world/2019/aug/25/asylum-seekers-limbo-eu-countries]

9. UNICEF. More than 1100 unaccompanied refugee and migrant children in Greece need urgent shelter and protection. United Nations International Children's Emergency Fund (UNICEF); 2019. [Available from: https://www.unicef.org/press-releases/more-1100-unaccompanied-refugee-and-migrant-children-greece-need-urgent-shelter-and]

10. AIDA, ECRE. The concept of vulnerability in European asylum procedures. Asylum Information Database (AIDA) and European Council on Refugees and Exiles (ECRE); 2017. [Available from: http://www.asylumineurope.org/sites/default/files/shadow-reports/aida_vulnerability_in_asylum_procedures.pdf]

11. Galos E, Bartolini L, Cook H, Grant N. Migrant Vulnerability to Human Trafficking and Exploitation: Evidence from the Central and Eastern Mediterranean Migration Routes. International Organization of Migration (IOM), Genevea; 2017. [Available from: https://publications.iom.int/system/files/pdf/migrant_vulnerability_to_human_trafficking_and_exploitation.pdf]

12. EKKA. Situation Update: Unaccompanied Children (UAC) in Greece National Center for Social Solidarity (EKKA), Greece; 2018. [Available from: https://www.unicef.org/eca/sites/unicef.org.eca/files/2018-12/Unaccompanied%20children%20in%20Greece%20%2030%20nov%202018.pdf]

13. EKKA. Situation Update: Unaccompanied Children (UAC) in Greece. National Center for Solidarity (EKKA), Greece; 2019. [Available from: https://www.unicef.org/eca/sites/unicef.org.eca/files/2019-01/Unaccompanied%20children%20in%20Greece%20Jan%202019.pdf]

14. UNHCR, UNICEF, IOM. Refugee and migrant children in Europe: Accompanied, unaccompanied, and separated. United Nations High Commisioner for Refugees (UNHCR), United Nations International Children's Emergency Fund (UNICEF), and the International Organization of Migration (IOM); 2017. [Available from: http://migration.iom.int/docs/Infographic_on_Children__Q3_2017_.pdf]

15. Brun D. Men and boys in displacement: Assistance and protection challenges for unaccompanied boys and men in refugee contexts. Care International; 2016. [Available from: https://reliefweb.int/sites/reliefweb.int/files/resources/CARE-Promundo_Men-and-boys-in-displacement_2017.pdf]

16. Kotef H. Objects of Security: Gendered Violence and Securitized Humanitarianism in Occupied Gaza. Comparative Studies of South Asia, Africa and the Middle East. 2010;30(2):179-91.

17. Fassin D. Inequality of Lives, Hierarchies of Humanity. In: Feldman I, Ticktin M, editors. In the Name of Humanity: The Government of Threat and Care. Durham, NC: Duke University Press; 2010.

18. Ticktin M. Casualties of care: Immigration and the politics of humanitarianism in France. Berkeley: University of California Press; 2011.

19. Collyer M. In-Between Places: Trans-Saharan Transit Migrants in Morocco and the Fragmented Journey to Europe. Antipode. 2007;39(4):668-90.

20. Schapendonk J. Migrants' Im/Mobilities on Their Way to the EU: Lost in Transit? Tijdschrift voor economische en sociale geografie. 2012;103(5):577-83.

21. Kaytaz ES. Afghan Journeys to Turkey: Narratives of Immobility, Travel and Transformation. Geopolitics. 2016;21(2):284-302.

22. Mainwaring Ċ, Brigden N. Beyond the Border: Clandestine Migration Journeys. Geopolitics. 2016;21(2):243-62.

23. Monsutti A. Migration as a Rite of Passage: Young Afghans Building Masculinity and Adulthood in Iran. Iranian Studies. 2007;40(2):167-85.

24. Markstrom CA, Iborra A. Adolescent Identity Formation and Rites of Passage: The Navajo Kinaaldá Ceremony for Girls. Journal of Research on Adolescence. 2003;13(4):399-425.

25. Vigil JD. Street Baptism: Chicano Gang Initiation. Human Organization. 1996;55(2):149-53.

26. Delaney CH. Rites of passage in adolescence. Adolescence. 1995;30(120):891.

27. Dunham RM, Kidwell JS, Wilson SM. Rites of Passage at Adolescence: A Ritual Process Paradigm. Journal of Adolescent Research. 1986;1(2):139-53.

28. McDade TW, Harris KM. The Biosocial Approach to Human Development, Behavior, and Health Across the Life Course. RSF. 2018;4(4):2-26.

29. WHO. Adolescence: Psychological and Social Changes. World Health Organization (WHO); 2014. [Available from: http://apps.who.int/adolescent/second-decade/section2/page5/adolescence-psychological-and-social-changes.html]

30. Sanders RA. Adolescent Psychosocial, Social, and Cognitive Development. Pediatrics in Review. 2013;34(8):354.

31. Elder G. Perspectives on the life course. In: Elder G, editor. Life Course Dynamics. Ithaca: Cornell University Press; 1985.

32. Elder G. The Life Course as Developmental Theory. Child development. 1998;69(1):1-12.

33. Sampson RJ, Laub JH. A life course theory of cumulative disadvantage and the stability of delinquency. Developmental theories of crime and delinquency. Advances in criminological theory. Piscataway, NJ, US: Transaction Publishers; 1997. p. 133-61.

34. Moffitt TE. Adolescence-limited and life-course-persistent antisocial behavior: a developmental taxonomy. Psychological review. 1993;100(4):674-701.

35. Magnani R, Sabin K, Saidel T, Heckathorn D. Review of sampling hard-to-reach and hidden populations for HIV surveillance. AIDS (London, England). 2005;19 Suppl 2:S67-72.

36. Nelson IA. From Quantitative to Qualitative: Adapting the Life History Calendar Method. Field Methods. 2010;22(4):413-28.

37. Schwarz I. Migrants moving through mobility regimes: The trajectory approach as a tool to reveal migratory processes. 2018 03/16. Report No.: 0016-7185. [Available from: https://doi.org/10.1016/j.geoforum.2018.03.007]

38. Jakobsen M, Meyer DeMott MA, Wentzel-Larsen T, Heir T. The impact of the asylum process on mental health: a longitudinal study of unaccompanied refugee minors in Norway. BMJ open. 2017;7(6):e015157.

39. Keles S, Idsoe T, Friborg O, Sirin S, Oppedal B. The Longitudinal Relation between Daily Hassles and Depressive Symptoms among Unaccompanied Refugees in Norway. Journal of abnormal child psychology. 2017;45(7):1413-27.

40. Feldman I. Care and Suspicion: Corruption as Definition in Humanitarian Relations. Current Anthropology. 2018;59(S18):S160-S70.
