# Supplementary material for: Interpretation of vulnerability and cumulative disadvantage among unaccompanied adolescent migrants in Greece: A qualitative study
Source: PLoS Med. 2020 Mar 27;17(3):e1003087. doi: 10.1371/journal.pmed.1003087 (PMC7100937; doi:10.1371/journal.pmed.1003087)
Supplement: Attachment — Submitted filename: Response to Reviewers.docx [file pmed.1003087.s006.docx]

Responses to Editors and Reviewers

# **Responses to comments from the Editors:**

Please revise your title according to PLOS Medicine's style. Your title must be nondeclarative and not a question. It should begin with main concept if possible. "Effect of" should be used only if causality can be inferred, i.e., for an RCT. Please place the study design ("A randomized controlled trial," "A retrospective study," "A modelling study," etc.) in the subtitle (ie, after a colon).

The title has revised to “*Interpretation of vulnerability and cumulative disadvantage among unaccompanied adolescent migrants in Greece”* to center the main concepts of the paper.

Please ensure all questionnaires in relation to interview questioning are provided either as citation of published interviews or submitted as Supp Files.

Interview guides have been submitted as the following supplementary files:

“S1 Text” for Key Informant Interview Guide

“S2 Text” for In-depth Interview Guide

“S3 Text” for Follow-up Interview Guide

Abstract: please specify dates with months as well as years; please provide some summary demographic information; Please use ‘youths’ instead of ‘youth’.

Line 24 has been updated to reflect months as well as years: “Between August 2018 and April 2019”.

Summary demographic information has been provided in lines 27-28: “44 male migrant youths from Afghanistan, Pakistan, Bangladesh, and Iran”.

The word “youth” has been changed to “youths” throughout the manuscript where it refers to more than one individual.

We appreciate the data cannot be made available, for the reasons you outline. You provide 2 email addresses for researchers who may be eligible to access data via contacting these persons. Note Paul Spiegel cannot be a point of contact as he is an author. Please delete. The remaining contact is fine if it isn’t an author.

Paul Spiegel has been removed as a point of contact. Contact information for Makieba Duff ([mduff4@jhu.edu](mailto:mduff4@jhu.edu)), who is not an author, has been included instead.

At this stage, we ask that you include a short, non-technical Author Summary of your research to make findings accessible to a wide audience that includes both scientists and non-scientists. The Author Summary should immediately follow the Abstract in your revised manuscript. This text is subject to editorial change and should be distinct from the scientific abstract. Please see our author guidelines for more information: <https://journals.plos.org/plosmedicine/s/revising-your-manuscript#loc-author-summary>

An Author Summary has been submitted.

Refs in main text – please use square instead of round brackets.

Round brackets have been replaced with square brackets throughout the manuscript.

Line 107 – please define “holistic case management services”

Holistic case management services have been specified in what are now lines 120-122: “…provided holistic case management services until the youths were successfully placed, including legal support, mental health services, accompaniment to medical appointments, and educational activities with peer groups”.

Did your study have a prospective protocol or analysis plan? Please state this (either way) early in the Methods section.

In the beginning of the Methods section, lines 112-113 state that the study is primary exploratory in nature, as is common among qualitative studies that use combined deductive and inductive analysis methods (see more detail on this below).

Please provide table in the main text of the demographic information re the boys included in the study: country of origin, age when arrived in Greece and other information.

Table 1 on page 8 has been added to give an overview of boys’ demographic information, including nationality, age at interview, age at departure from home country, whether or not they were in children’s shelters before age 18, and their housing situation at the time of the interview.

a) If a prospective analysis plan (from your funding proposal, IRB or other ethics committee submission, study protocol, or other planning document written before analyzing the data) was used in designing the study, please include the relevant prospectively written document with your revised manuscript as a Supporting Information file to be published alongside your study, and cite it in the Methods section. A legend for this file should be included at the end of your manuscript.

b) If no such document exists, please make sure that the Methods section transparently describes when analyses were planned, and when/why any data-driven changes to analyses took place.

No such document exists. However, the Methods section has been updated in 170-175 to transparently state which categories for thematic coding were defined a priori (deductive analysis) and where codes were derived inductively in accordance with themes that emerged from the data. A paper on applied thematic analysis (ref 44.) is cited in this section in case readers are interested in an overview of inductive (exploratory) and deductive (confirmatory/determined in advance) strategies of coding in qualitative data analysis.

c) In either case, changes in the analysis—including those made in response to peer review comments—should be identified as such in the Methods section of the paper, with rationale.

Please provide a reporting checklist – perhaps COREQ <https://www.equator-network.org/reporting-guidelines-study-design/qualitative-research/?post_type=eq_guidelines&eq_guideli>

The reporting of the methods has been updated according to COREQ (pages 6-10), which was a very helpful suggestion. We intended to have a strong inductive component from the outset, as explained in part (b) of this comment and stated in lines 170-175.

No changes to analysis were made in response to peer review comments.

# **Responses to Reviewer 1:**

This is a statistical review of manuscript PMEDICINE-D-19-03759. This manuscript describes the results of interviews and life history calendars of 44 male migrant youth who arrived in Greece as unaccompanied minors.

The research methods are mostly qualitative. Life-course theory belongs to the social sciences. Nonetheless, from a statistical perspective, some clarifications would improve the manuscript. My comments are listed below.

Methods line 114: 9 staff members from three organizations were interviewed. I suggest that you clarify how many were interviewed by organization. Is it one staff member for two of the organisations, and 7 from the third organisation, or it is more balanced?

The number of staff from each organization have been specified in lines 123-127:

“Two lawyers, a social worker, a psychologist, and a cultural mediator were interviewed at NGO X. NGO Y operated one specialized shelter for UAMs, transferred UAMs from the islands to the mainland, provided legal guardians for UAMs, and referred them to other shelters when needed. A social worker and project manager were interviewed at NGO Y. Transitional Shelter Z was staffed by a social worker and psychologist, both of whom were interviewed.”

Methods line 117: could you please explain your sample size? Why did you chose 44 youth? Is it due to logistical/feasibility reasons, or did you "calculate" that with 44 youth your interviews would be sufficiently "representative"?

The sample size was determined by thematic saturation. This has been clarified in lines 136-141:

“Recruitment continued until thematic saturation was reached at 44 interviews [40]. Thematic saturation depended, in turn, on including sufficient number of study participants from the two main source countries for UAMs in Greece, which were Afghanistan and Pakistan [12], sufficient number of study participants placed in children’s shelters, and sufficient number of participants who were not placed in children’s shelters. This could only be determined as the study proceeded, rather than prospectively [41, 42]”.

Recent review of the issues involved in determination of sample size in qualitative studies are found in:

- Weller SC, Vickers B, Bernard HR, Blackburn AM, Borgatti S, Gravlee CC, et al. (2018) Open-ended interview questions and saturation. PLoS ONE 13(6): e0198606. https://doi.org/ 10.1371/journal.pone.0198606
- Malterud, K., Siersma, V. D., & Guassora, A. D. (2016). Sample Size in Qualitative Interview Studies: Guided by Information Power. Qualitative Health Research, 26(13), 1753–1760. https://doi.org/10.1177/1049732315617444

Methods line 124: please could you explain what snowball sampling is for the reader? In particular, could you provide the total pool of potential UAMs in each subgroup, and how many you interviewed per subgroup?

Lines 134-135 explain snowball sampling in greater detail: “A snowball sampling strategy was used in which seeds were recruited from each social group to both participate in interviews and help identify other eligible youths for recruitment [36]”.

In lines 133, the word “subgroups” has been updated to “social groups”, which we have determined to be more accurate, and lines 133-134 specify that social groups were “largely defined by nationality, history in children’s shelters, and current living arrangements.”

Table 1 on page 7 enumerates participants’ nationality, history in children’s shelters, and current living arrangements.

Introduction line 57: Greece hosts *an* estimated 32,000…

This has been corrected in lines 60.

# **Responses to Reviewer 2:**

This is a very interesting analytical piece on the vulnerabilities and cumulative disadvantage for unaccompanied adolescent migrants (UAMs) in Greece. It is highly relevant to the ongoing debate on the need to facilitate the social protection of refugees and migrants, particularly children and adolescents. However, the paper would benefit from a more detailed description and in-depth analysis of potential factors that have led to the vulnerability of male UAMs. It would also benefit from a comparative approach to assessing the experiences of interviewed UAMs.

1. The research question is an important one to the community of researchers in this general area

Studying the effect of displacement on asylum seekers is important in the context of population displacement due to armed conflict. Among these population, the most vulnerable subgroup is children, especially those with no family or companionship. The study is worth publishing as it assesses vulnerable groups who lack social support.

Thank you.

2. The results provide a substantial advance over existing knowledge, with clear implications for patient care, public policy, or clinical research agendas

The results of this study can be used to guide public policies and international organizations proving relief and aid for disadvantaged un-accompanies minors and children, as well as adults in host countries.

Thank you.

3. Published together with an Author Summary written for general readers, the article is of interest to clinicians and policymakers who are not specialists in this topic

This paper discussed an important topic that would be of interest to PLOS Medicine readership.  I did not have access to the Author Summary written for general readers.

Thank you for these comments. We have now updated the Author Summary as a separate file.

4. What are the main claims of the paper and how significant are they for the discipline?

This study is important since it reports on disadvantage subgroups among a vulnerable population. The study has shown that children who are not placed in shelters become more disadvantaged. It also shows that age is not the only factor to be considered when assessing vulnerability, cumulative disadvantages such as current living conditions, work, and other factor are just as important. The findings of this paper can help guide international organization and other aid programs in improving the relief efforts and maximizing efficiency of the aids targeting this population.

Operationalizing vulnerability in the paper is based only on the views of some representatives from three organizations. It would be helpful to provide more details on this concept (e.g., a lawyer was interviewed, so what are the legal issues concerning operationalizing vulnerability).

It would also be helpful to provide more insights on the shortcomings of how the organizations operationalize vulnerability and explicitly list the main themes that emerged (e.g., focus on age, etc.). The themes could be organized din subheadings to guide the readers.

In response to these comments, the wording in the results section has been refined to focus on how vulnerability is interpreted and used in the bureaucratic process of referring migrant youths to shelters.

To guide readers, the themes that emerged from key informant interviews have been organized into three subheadings in lines 195-221. These are “Differing perceptions of vulnerability” (line 195), “Variability in reporting vulnerability” (line 210), and “The importance of age” (line 221).

The shortcomings of how vulnerability is interpreted by organizations is discussed in the Discussion section in lines 344-346.

5. Are the claims properly placed in the context of the previous literature? Have the authors treated the literature fairly?

Although the authors refer to the life course theory in the introduction and the objectives, further details on how the theory was used are missing. Links to the theory should be provided.

The literature review can benefit from the addition of a number of recent articles addressing the topic. A number of recent articles assess the experiences of displaced unaccompanied minor refugees in Europe have been missed.

Examples of recent studies on unaccompanied minors:

a. Ferrara, P.; Corsello, G.; Sbordone, A.; Nigri, L.; Caporale, O.; Ehrich, J.; Pettoello-Mantovani, M. The "Invisible Children": Uncertain Future of Unaccompanied Minor Migrants in Europe. The Journal of Pediatrics 2016, 169, 332-333.e331, doi:<https://doi.org/10.1016/j.jpeds.2015.10.060>.

b. Pradella, F.; Pinchi, V.; Focardi, M.; Grifoni, R.; Palandri, M.; Norelli, G.A. The age estimation practice related to illegal unaccompanied minors immigration in Italy. The Journal of forensic odonto-stomatology 2017, 35, 141-148.

c. Abbing, H.D. Age determination of unaccompanied asylum seeking minors in the European Union: a health law perspective. European journal of health law 2011, 18, 11-25, doi:10.1163/157180911x546101.

Thank you for these suggestions. Ferrara (2016) is now cited in line 66. Pradella (2017) and Abbing (2011) have not been included as their focus, while important, does not directly relate to the topic of the paper. They focus on age estimation/determination, which is an important issue and a process that typically occurs soon after an unaccompanied minor arrives, usually on the islands. This paper, on the other hand, focuses on youth whose identity as minors was already established, and examines their life trajectories into adulthood.

Additionally, the following articles are also referenced in the updated Introduction section (line 66):

- Freccero J, Biswas D, Whiting A, Alrabe K, Seelinger KT. Sexual exploitation of unaccompanied migrant and refugee boys in Greece: Approaches to prevention. PLoS medicine. 2017;14(11):e1002438.
- Digidiki V, Bhabha J. Sexual abuse and exploitation of unaccompanied migrant children in Greece: Identifying risk factors and gaps in services during the European migration crisis. Children and Youth Services Review. 2018;92:114-21.

6. Do the data and analyses fully support the claims? If not, what other evidence is required?

The analysis of the qualitative data is supportive of the claims presented in this paper.

However, the way the results were presented could be improved. The authors say that: "the following sections follow the experiences of three youths whose experiences were representative of the 44 study participants " .

I suggest to omit the word 'representative' since this is a qualitative study that mainly narrates the experience of three male youth.

The word “representative” has been omitted. In its place, the introduction to the case studies now reads (lines 294-295) “This section follows the experiences of three youths as case studies to illustrate how the accumulation of disadvantages shapes their trajectories and how children’s shelters can alter them.”

One thing that would make the paper stronger is a bit more elaboration on the issue of determination of vulnerability. It seems to me that there is something important to say about the process through which the youth are evaluated, referred and then characterized as more or less vulnerable. Reading the first subsection of the "Results" section gives the impression that part of the problem could be in how some NGO staff represent the children's cases to the EKKA, for example "[The case worker] may write one or two sentences, that [the UAM] is homeless, that he is distressed. [This] vague, general information doesn't help the boy." (page 9, lines 179-80). Or that the problem may be an issue in the procedure, the forms that the NGOs send and the kind of information requested in these forms. Are these forms prepared by the EKKA? Do they need to add more informative questions or sections where the NGO workers can include a bit of the history of the children and their current work/living conditions?

What are the recommendations of the authors to improve how vulnerability is operationalized? How can the cumulative vulnerability be taken into account when assessing the needs of the UAMs?

These are very insightful comments and have been addressed in the updated Discussion section. Recommendations have been added in lines 412-417, including the addition of items on the referral form:

“Structured reporting of certain parameters in the referral forms distributed by EKKA, such as the duration of youths’ journeys, histories of education, and child labor, may encourage systematic prioritization of the most vulnerable UAMs instead of relying on variable reporting by NGO staff. Keeping in mind the long-term consequences for youth who are not placed in shelters before they turn 18, 17-year-old youth from significantly disadvantaged backgrounds should be prioritized as urgent cases for protection, or targeted by other programs that facilitate integration after they turn 18.”

It has also been specified in lines 188 and 513 that the referral forms are distributed by EKKA.

7. PLOS Medicine encourages authors to publish detailed methods as supporting information online. Do any particular methods used in the manuscript warrant such publication? If a protocol is already provided, for example for a randomized controlled trial, are there any important deviations from it? If so, have the authors explained adequately why the deviations occurred?

The interview guides were not provided by the authors.

The interview guides have been submitted as supplementary files alongside the revised manuscript.

8. Is this paper outstanding in its discipline? If yes, what makes it outstanding? If not, why not?

This paper helps guide the aid efforts of international and local organization to improve the conditions of vulnerable and unaccompanied minors.

Thank you.

9. Are details of the methodology sufficient to allow the experiments to be reproduced?

It would be helpful to clarify the rationale for selecting only three out of 44 male UAMs in order to reflect in detail on their experiences since a larger and more varied group was not selection.

In the first paragraph of the methods (line 103-116), the authors did not specify how and why the 2 NGOs and Shelter were identified and selected for the study. Also since the interviews were not in English, did they translate them? This was not mentioned. In addition to interview guides were not provided.

Themes from the more varied group of 44 UAMs have been presented under the subheadings “Disadvantages prior to departure” (line 245) and “Disadvantages as an irregular child migrant” (line 254), as well as in Table 2 and Table 3. The introductions to the three case studies has been updated to say that they were selected because they “illustrate how the accumulation of disadvantages shapes [UAMs’] trajectories and how children’s shelters can alter them” in lines 294-295.

The Methods section has been updated to reflect that the NGOs were purposively selected in lines 115-118:

“NGOs were purposefully selected due to their prominence in child protection efforts in Athens and their close relationships with the Greek government and UNHCR. Transitional Shelter Z was selected because its work with former UAMs offered key insights into unaccompanied migrant youths’ transition into adulthood.”

Key informant interviews were in English, but interviews with migrant youth were not. The methods section has been updated to clarify that in-depth interviews were transcribed and translated into English in line 154-155:

“All interviews were audio-recorded, then transcribed and translated into English.”

Interview guides have been submitted as supplementary files.

10. Is the manuscript well organized and written clearly enough to be accessible to non-specialists?

The author in the background and methods section explained most of the terms and concepts used in this paper, so that readers can follow and understand the findings of this paper. However, It would be helpful to refer to a definition of 'cumulative disadvantage' as was the case for vulnerability.

As for the organization, the overall paper is well organized, however the results section could have been better presented, especially the tables. The flow of the information in the results can be improved by separating the sections on the general results (of the 44 youths) and those of the three youths.

Spelling and grammar mistakes were also identified in this paper.

Thank you. Cumulative disadvantage has been defined in line 101: “…cumulative disadvantage refers to cycles of reciprocal interactions between an individual’s reaction to his or her disadvantage and the environment’s response to his reaction, which cause disadvantage to accumulate over years and lead to poor outcomes in adulthood.”

In response to these comments, the Results section has been reorganized into the following 3 subsections: “Interpretations of vulnerability” (lines 186-243) which only reports findings from key informant interviews, “Cumulative disadvantages” (lines 244-292), which reports themes regarding disadvantages from the sample of 44 youth, and “Case studies” (lines 293-332), which have been separated out from the more general sections.

11. Other comments

1)Title:

Please revise the title. The use of "neither" is followed by "nor". For example: Neither minors nor adults.

Make sure to use either singular or plural: minors/adults or minor/adult.

"Interpretation of vulnerability and cumulative disadvantage":

This sentence reads as interpretation of vulnerability and interpretation of cumulative disadvantage. The way the short title is worded is better. Another option is to reverse the title:

Cumulative disadvantage and interpretation of vulnerability for unaccompanied adolescent migrants in Greece.

In light of these comments, the title has been updated to “Interpretation of vulnerability and cumulative disadvantage among unaccompanied adolescent migrants in Greece”.

2) ABSTRACT:

"Operationalization of vulnerability":

Operationalization is different than interpretation. The first follows a specific, agreed-upon set of criteria or rules whereas the second is a matter of personal evaluation and assessment. The two terms should not be used interchangeably. This is important in order to clearly frame and present the issue or problem presented in this paper.

The wording has been changed to focus only on interpretation (line 22).

"Due to the way vulnerability was interpreted and operationalized by the shelter system for UAMs---":

This sentence needs clarification. What is the consequence of the shelter system's interpretation of vulnerability?

Is it:

a) that youth who requested shelter... had the greatest need to learn new skills to facilitate their integration?

Or

b) that youth who had the had the greatest need to learn new skills to facilitate their integration often had the least opportunity to do so?

The Conclusions section has been clarified to say that “Due to the way vulnerability was interpreted by the shelter system for UAMs, youths who had the greatest need to learn new skills to facilitate their integration often had the least opportunity to do so” in lines (43-45).

3) Other potential limitation is restricting the sample to male UAMs. This was not mentioned in the limitations, only in the introduction of the manuscript.

The limitations section has been updated to note that results may not be applicable for female UAMs (line 406-407):

“…the results of this study may not be applicable to female UAMs, as their experiences along clandestine journeys and with NGOs may differ significantly from males.”

4) In the Background section, the author could talk more about the type of relief efforts targeted for this vulnerable population in general.

The type of relief efforts provided vary drastically from shelter to shelter. At some shelters, unaccompanied minors are enrolled in schools while in others, they do not even get access to Greek or English lessons. Other provide activities to build peer groups, such as soccer games, while in others, children are neglected and incidents of fights and self-harm are high. Most shelters employ staff to provide mental healthcare but this too variable. For the purposes of this paper, we added a reference to the variability of services provided in shelters in line 67-68:

“NGOs in Greece operate shelters for unaccompanied children, which provide psychosocial services of variable kind and quality to facilitate their integration.”

5) Line 103: replace "from" by "with"

This has been replaced. See line 110.

6) Line 212: replace "They family" by "Their families"

 “They family did not own property” has been changed to “They did not own property”. See line 302.

7) In table 2, Under Bilal: replace "whereas his father's side of the was implicated" by "whereas his father's side was implicated"

This has been replaced. See Table 4, under Bilal.

8) Table 3, in the row on "In the care of relatives": replace: "differences" by "differences in"

This has been replaced. See Table 3, in the row on "In the care of relatives".

9) Table 4, in the first column, in the last paragraph: replace "he savings" by "his savings"

This has been replaced. See Table 5, first column.

10) Table 4, in the third column, in the last paragraph: replace "to arrive in Greece" by  "to arrive to Greece"

This has been replaced with “to reach Greece”. See Table 5, third column.

11) Line 281: replace "he could not to apply" by "he could not apply"

This has been replaced. See line 329.

12) Line 281: what do you mean by "sold sex"? Please consider rephrasing

This has been replaced with “engaged in transactional sex to survive.” See line 331.

13) Table 5, first column: "he not been able to find a job" by "he hasn't been able to find a job"

This was replaced with “he wasn’t able to find a job”. See Table 6, first column.

14) Table 5, second column, last paragraph: replace "transferred to a shelter" by "transferred him to a shelter".

This has been replaced. See Table 6, second column.

15) In table 5, there is a sentence |See chapter 2 for details" - Where is chapter 2?

This was erroneous and has been removed.

16) Line 319: replace "this is illustrated the trajectory" by "this is illustrated by the trajectory"

This statement has been deleted completely due to the restructuring of the results section.

17) Line 335: replace "which exposed them exploitive circumstances" by "which exposed them to exploitive circumstances"

This has been replaced. See line 374.

18) Table 1 and 2 has the same title (maybe the author could merge them together).

All tables have been given distinct, more informative titles and case study tables have been numbered.

What was formerly Table 1 is now Table 2, Disadvantages Prior to Departure. What was formerly Table 2 is now Table 3, Circumstances Experiences While Living Alone.

19) I suggest putting table 4 before table 3 (and their corresponding paragraphs), this way it is more in line with the timeline: first their experience in transit to Turkey or Iran and then their experience living alone.

This has been changed, and the tables presenting the three case studies now come one after another as Table 4, Table 5, and Table 6.

20) The titles of all the table could be improved. For example: comparative case studies, part 3 - what does this mean? Where is part 1 and 2?

All tables have been given distinct, more informative titles and case study tables have been numbered. Tables have the following names:

Table 1. Participant Demographics

Table 2. Disadvantages prior to departure

Table 3. Circumstances experienced while living alone

Table 4. Comparative case studies, part 1: Home countries

Table 5. Comparative case studies, part 2: Journeys

Table 6. Comparative case studies, part 3: Greece

21) Also the tables could be better presented for organization and visual purposes.

We agree and have reformatted tables to have no shading in the backgrounds, and Tables 4-6 now have a horizontal orientation.

22) In line 198 to 200, why are tables 1 and 3 not explained (as what to do they present) as the rest of the tables?

This issue has been eliminated with the overall restructuring of the results section.

# **Responses to Reviewer 3**

Line 54: The data for UAM’s in Greece needs to be updated to reflect numbers in 2018 and 2019 when the research was carried out. There are weekly updates at: <https://data2.unhcr.org/en/documents/download/72825>

Lines 60-61 reflect numbers in 2019.

Line 55: Definitely necessary to mention the EU-Turkey agreement here, which is what has had the most impact on the migration situation in Greece and the ‘hotspots’ approach which was implemented subsequently and has resulted in the resultant containment of asylum seekers

Lines 58-59 have been updated to reference the EU-Turkey agreement.

Line 95: His or her?

Line 102 has been updated to include both pronouns.

Line 97: A definition of both UAMs and adolescence would be helpful at some point prior to their use in the article.

UAMs have been defined in lines 54-55 of the revised draft:

“In Europe, the number of unaccompanied minors (UAMs), or migrants under the age of 18 traveling without adult family members, spiked from 13,800 in 2013 to 23,300 in 2014…”

Adolescents have been defined in lines 82-83:

“the World Health Organization defines adolescence as ages 10-19 [20].”

Line 113: Unclear how sampling was carried out and why different numbers of staff were interviewed at each of the locations

Lines 115-120 have been updated to clarify that NGOs were purposively selected and the number of staff interviewed at each organization varied in accordance to the size and structure of the different organizations:

“NGOs were purposefully selected due to their prominence in child protection efforts in Athens and their close relationships with the Greek government and UNHCR. Transitional Shelter Z was selected because its work with former UAMs offered key insights into unaccompanied youths’ transition into adulthood. The number and type of staff interviewed at each organization varied by the size and structure of the organizations.”

Line 126: Please outline if these were conducted by the PI, or whether there was a translator

Lines 147-151 have been updated to specify that interviews were conducted by author DM with the assistance of interpreters:

“Interviews were conducted by author DM, who had volunteered as an Urdu interpreter on Lesbos island in 2016, and since maintained relationships with Greece’s Afghan and Pakistani migrant communities. Interviews were conducted with the support of trained interpreters from migrant communities in Farsi, Dari, or Urdu to target Afghan and Pakistani youths as they represent the most common nationalities among UAMs in Greece.”

Line 130: Please explain the ethics review process that was undertaken: both at the academic institution of the author, and via any of the NGOs who participated.

The ethics process was undertaken at Johns Hopkins School of Public Health. NGOs were not involved in ethics reviews as staff were only interviewed regarding their professional work, and none of the NGOs were involved in recruitment of migrant youth. This stated in lines 178-180.

Line 196: It would be good to explain how these are representative here i.e. is this because of the types of disadvantages these boys faced were shared by the others; are there numbers/an overview table of how many of the remaining of the 44 participants experienced the same disadvantages?

Table 1 (after line 144) has been added to provide demographic and other relevant data regarding the sample of 44 youths, including access to children’s shelters, age at departure from home country, and housing situation at the time of interview.

Table 2: It would be good to have some further comment/reflection on these case studies after this table

This has been addressed by restructuring the Results section so that there is a subsection entirely devoted to discussing case studies.

Line 264: How representative was this however? It would be good to have some overview of the interview topics and how often these came up at some point in the results section

The term “representative” has been removed and the introduction of the case studies has been revised to state that they illustrate key points rather than represent the sample of 44. See lines 294-295:

“This section follows the experiences of three youths as case studies to illustrate how the accumulation of disadvantages shapes their trajectories and how children’s shelters can alter them.”

Interview guides have been submitted as supplementary files so that the topics discussed are readily available.

Line 278: Again, please represent in the results section how representative this was of the 44 interviews (i.e. how many of them had similar experiences in terms of integration)

The term “representative” has been removed and the introduction of the case studies has been revised to state that they illustrate key points rather than represent the sample of 44. See response to previous comment.

Line 286: Important to link back to the ideas around ‘vulnerability’ and the fact that migrant men are not perceived as vulnerable

With restructuring of the Results section, this information is now placed in the “Interpretations of vulnerability” section in lines 186-243.

Line 294: Overall comment on results section: It would be good to see an overview of all the results at the beginning of the results section. For example, I didn’t feel that I had insight into the results of the interviews with the service providers at the NGOs. It would be good to have more of an overview of both the interviews with adolescents as well as with the NGOs at the beginning of the results section - in terms of the main themes

An overview of the results has been added in lines 182-185:

“This section first presents interpretations of vulnerability identified in key informant interviews, followed by an overview of disadvantages faced by youths in their home countries and during their journeys. Then, three case studies are presented to illustrate accumulating disadvantages over UAMs’ lifetimes and the pivotal role of children’s shelters in shaping life trajectories.”

Line 295: This wasn’t made fully clear by the results that were presented in the results section

We have removed line 295 of the initial submission.

Line 305: There needs to be a clearer linkage in the discussion section - with the sub-headings of the results section.

Thank you for this comment. The Discussion section has been reorganized (pages 26-30) to follow the progression of the updated results section, which is now laid out in the overview in lines 182-185.

Line 343: I didn’t see the results from the life history calendars integrated into the results section

This line has been reworded to refer to the use of life course theory and cumulative disadvantage as analytical lenses in line 389-391:

“The use of life course theory and cumulative disadvantage as analytical lenses [36, 37] allowed for an understanding of how disadvantages in UAMs’ home countries can lead to prolonged journeys [38], and shape the kinds of experiences UAMs’ have during prolonged journeys.”
