# Supplementary material for: Interpretation of vulnerability and cumulative disadvantage among unaccompanied adolescent migrants in Greece: A qualitative study
Source: PLoS Med. 2020 Mar 27;17(3):e1003087. doi: 10.1371/journal.pmed.1003087 (PMC7100937; doi:10.1371/journal.pmed.1003087)
Supplement: Attachment — Submitted filename: Response to Reviewers 3.11.docx [file pmed.1003087.s007.docx]

Production Requirements

TABLES and BOXES:

1. Table 4 uses returns to control text wrapping. This is indicated by a [¶ or ↵] symbol when "Show Paragraph Marks" is turned on in Word. Please either split those cells into separate cells to achieve alignment or remove the returns.

Hard returns have been removed in Table 4 and replaced with split cells.

REFERENCES:

1. Please put all reference citations in square brackets separated by either commas or dashes with no spaces, e.g. [1,2,3] or [1-3].

Square brackets have been used for all in-text citations. In text references to tables and supporting files have also been replaced with square brackets. If you still see any brackets inconsistent with these guidelines, could you please specify their location?

SUPPORTING INFORMATION:

1. Please ensure that all Supporting Information files use the following format verbatim throughout the manuscript (including legends and all in-text citations): S1 Fig, S1 Table, S1 Text, S1 Data, etc. Each file type should start at 1 and increase numerically. Please note, however, that the file names themselves must use an underscore rather than a space, i.e. S1_Fig.tif, S1_Table.xlsx, S1_Text.doc, S1_Data.xlsx, S1_PRISMA_Checklist.docx, etc.

The COREQ checklist has been renamed S1_COREQ_checklist. Otherwise I believe the manuscript is consistent with these guidelines. If you still see inconsistencies, could you please specify their location and the changes you would like? Thank you.
